# Association of Fungi and Archaea of the Gut Microbiota with Crohn’s Disease in Pediatric Patients—Pilot Study

**DOI:** 10.3390/pathogens10091119

**Published:** 2021-09-01

**Authors:** Agnieszka Krawczyk, Dominika Salamon, Kinga Kowalska-Duplaga, Tomasz Bogiel, Tomasz Gosiewski

**Affiliations:** 1 Department of Molecular Medical Microbiology, Faculty of Medicine, Jagiellonian University Medical College, 31-121 Krakow, Poland; agnieszka.krawczyk@doctoral.uj.edu.pl; 2 Department of Pediatrics, Gastroenterology and Nutrition, Faculty of Medicine, Jagiellonian University Medical College, 30-663 Krakow, Poland; kinga.kowalska-duplaga@uj.edu.pl; 3 Microbiology Department, Ludwik Rydygier Collegium Medicum in Bydgoszcz, Nicolaus Copernicus University in Torun, 85-094 Bydgoszcz, Poland; t.bogiel@cm.umk.pl; 4 Clinical Microbiology Laboratory, University Hospital No. 1 in Bydgoszcz, 85-094 Bydgoszcz, Poland

**Keywords:** fungi, archaea, gut microbiota, Crohn’s disease, gut dysbiosis

## Abstract

The composition of bacteria is often altered in Crohn’s disease (CD), but its connection to the disease is not fully understood. Gut archaea and fungi have recently been suggested to play a role as well. In our study, the presence and number of selected species of fungi and archaea in pediatric patients with CD and healthy controls were evaluated. Stool samples were collected from children with active CD (*n* = 54), non-active CD (*n* = 37) and control subjects (*n* = 33). The prevalence and the number of selected microorganisms were assessed by real-time PCR. The prevalence of *Candida tropicalis* was significantly increased in active CD compared to non-active CD and the control group (*p* = 0.011 and *p* = 0.036, respectively). The number of *Malassezia* spp. cells was significantly lower in patients with active CD compared to the control group, but in non-active CD, a significant increase was observed (*p* = 0.005 and *p* = 0.020, respectively). There were no statistically significant differences in the colonization by archaea. The obtained results indicate possible correlations with the course of the CD; however, further studies of the entire archeobiome and the mycobiome are necessary in order to receive a complete picture.

## 1. Introduction

**Figure 1:**
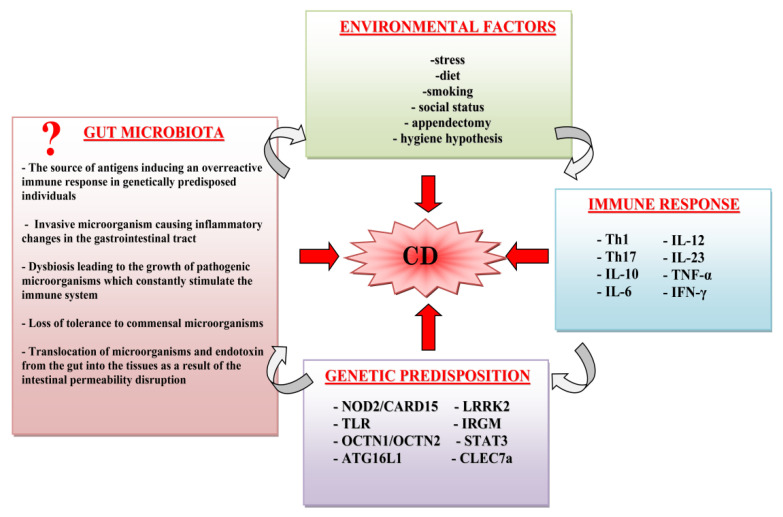
Risk factors for CD —Crohn’s Disease; NOD2—nucleotide-binding oligomerization domain containing 2; CARD15—caspase recruitment domain family, member 15; TLR—toll-like receptors; OCTN—organic cation transporter; ATG16L1—autophagy-related protein 16 like 1; LRRK2—leucine-rich repeat kinase 2; IRGM—immunity related GTPase M; STAT3—signal transducer and activator of transcription 3; CLEC7A—C-Type Lectin Domain Containing 7A, IFN-γ—interferon gamma, TNF-α—tumor necrosis factor alpha; Th1—Type 1 helper T-cells; Th17—T-helper 17 cell, Il- interleukin.

Crohn’s disease (CD) is included in the group of diseases known as inflammatory bowel diseases (IBD). It is believed that the incidence and severity of the course of the disease are conditioned by a combination of genetic, immunological, environmental and microbiological factors (Figure 1) [1,2,3]. The latter remain a constant interest of many research teams, due to the still unclear role of microorganisms in the pathogenesis of the disease. 

The fact that microorganisms participate in causing CD is suggested by numerous studies and clinical observations, among others: exacerbation of the disease as a result of infection and food poisoning [4]; failure to induce enteritis in germ-free animals; alleviation or amelioration of symptoms following the application of antibiotics, probiotics or colon cleansing treatments [5,6], as well as achieving remission in patients who underwent a transplant of gut microbiota from healthy people [7]. The majority of the studies to date have concerned the analysis of the bacteriobiome in IBD [8,9,10,11,12,13,14]. However, owing to the recent development of molecular methods, more and more attention is also paid to other microorganisms comprising the normal gut microbiota, i.e. fungi, viruses or archaea.

Fungi are organisms that belong to the domain *Eukaryota*. Despite the fact that they are a smaller population in the human microbiota than bacteria, their interactions with immune cells or the production of metabolites are relevant to human health and disease [15]. The studies to date indicate that the course of CD may be associated with the impact of mycobiome on GALT (gut-associated lymphoid tissue). The presence of antibodies against *Saccharomyces cerevisiae* (ASCA—anti-*Saccharomyces cerevisiae* antibodies) recognizing cell wall mannans of yeast is a clinical biomarker of patients with CD [16,17]. 

With regard to their general structure, archaea resemble bacteria, while genetically, they are more closely related to eukaryotes. They belong to the domain *Archaea*, among which three basic groups can be distinguished, which are halophilic, methanogenic and thermophilic archaea. Methanogens are the group that most frequently colonizes the human gastrointestinal tract. The importance of the archaea presence in the human gastrointestinal tract is not fully understood. Until now, strong emphasis has been placed on studying the role of bacteria in the course of CD, while the role of those microorganisms is so far less taken into account by researchers.

Therefore, the objective of this study was an assessment of the prevalence of the most common representatives of *Archaea* and yeast fungi in children and teenagers with CD at different stages of the disease. Moreover, the number of these microorganisms in individual groups was determined. 

## 2. Results

### 2.1. Characteristics of the Subjects

There were 124 children included in the study: 54 patients with active CD, 37 patients in remission and 33 healthy individuals for the control group. The age of each group of patients was similar: average 13.15 (±2.86) years for patients with active CD vs. 12.86 (±3.95) years for patients with non-active CD vs. 11.39 (±3.99) for healthy children from the control group. The group of patients with active CD consisted of 21 girls (39%). In the group of patients with non-active CD, there were 17 girls (46%). The control group included 19 girls (58%). The average Pediatric Crohn’s Disease Activity Index (PCDAI) value in the group of patients with active CD amounted to 33.23 pts (±13.30) while in the group of patients in remission, 3.7 pts (±3.65).

### 2.2. Prevalence of Fungi and Archaea in Stool Samples of Controls and Patients with CD

**Figure 2:**
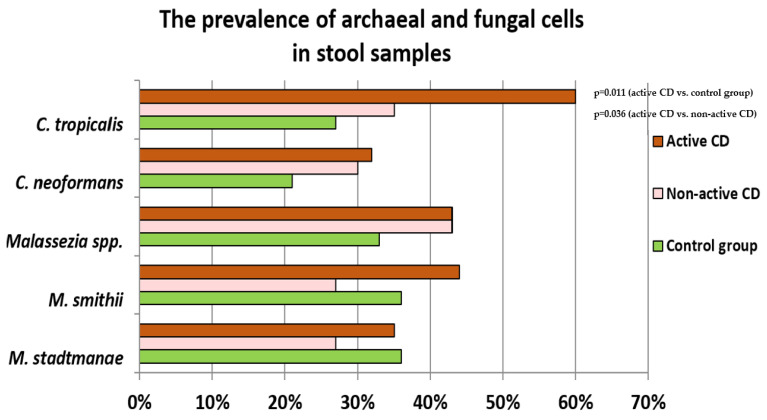
The percentage of positive samples for the respective archaea and fungi in the examined groups.

The presence of fungi and archaea were detected in the stool samples using the Real-Time PCR method. Candida tropicalis-positive samples were more frequent in the group of patients with active CD compared to non-active CD (*p* = 0.036) and control group (*p* = 0.011) (respectively 60% vs. 35% vs. 27%). Similar relationships were observed in the case of *Methanobrevibacter smithii* (respectively 44% vs. 27% vs. 36%), however, these changes were not statistically significant (*p* > 0.05). The prevalence of *Malassezia* spp. and *Cryptococcus neoformans* oscillated at a similar level in the group of patients with CD (*irrespective of disease activity*) and were slightly higher compared to the healthy control group (Figure 2), *p* > 0.05. The numbers of *Methanospharea stadtmanae*-positive samples were similar in the group of children with active CD and in the control group, while in patients with non-active CD, a somewhat lower prevalence of this methanogen was observed (Figure 2), *p* > 0.05.

Negative results were obtained for all negative controls in the assay and positive results were found in all positive controls.

### 2.3. The Number of Fungi Cells in Stool Samples 

**Figure 3:**
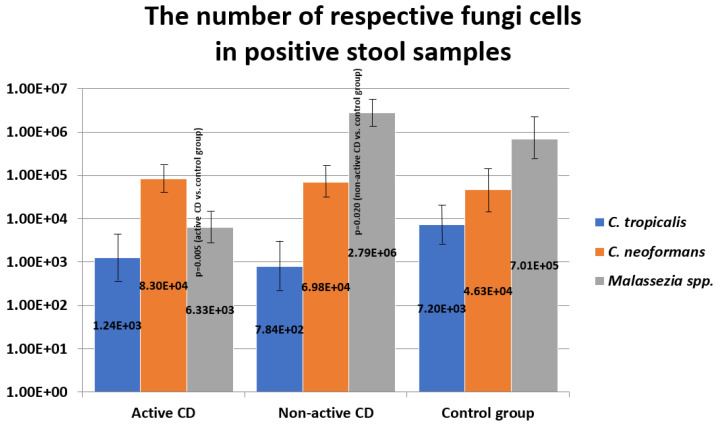
Number (fungal cells/g of stool) of fungi in the examined groups.

In positive samples, quantitative analysis was carried out in relation to the examined microorganisms. The mean number [fungal cells/g of stool] of fungi of the genus *Malassezia* was significantly lower in the group of children with active CD compared to the control group (*p* = 0.005). Whereas, among patients in remission, the number of *Malassezia* spp. cells increased and was significantly higher compared to the number of these fungi in the control group (*p* = 0.020). In regard to the mean numbers of *C. tropicalis* and *C. neoformans*, there were no significant differences between the groups (Figure 3). It was demonstrated that there is a low positive correlation between *C. tropicalis* and PCDAI (rho = 0.23; *p* = 0.031).

### 2.4. Methanogen DNA Concentration in Stool Samples

**Figure 4:**
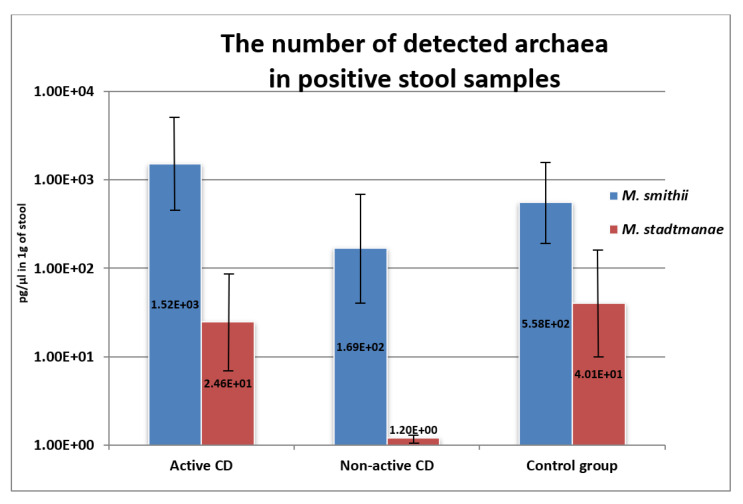
The number (pg/µL in 1 g of stool) of respective methanogens in the examined groups.

*M. smithii* DNA concentration [pg/µL DNA in 1 g of stool] was higher in the group of children with active CD compared to patients in remission or healthy individuals; however, these changes were not statistically significant (Figure 4). There were also no statistical differences concerning the numbers of *M. stadtmanae* between the groups, although the amount of methanogens tended to decrease along with the diminishing degree of disease activity (Figure 4). The correlation between the number of *M. smithii* and PCDAI was positive, but only weak and close to statistical significance (rho = 0.22, *p* = 0.051).

## 3. Discussion

Our study, using real-time PCR, determined the frequency of occurrence and abundance of three species of fungi (*C. tropicalis*, *C. neoformans* and *Malassezia* spp.) and two species of methanogenic archaea (*M. smithii*, *M. stadtmanae*) in children and adolescents with CD at different stages of the disease. The results of this study showed that patients in the active phase of the disease were found to demonstrate the presence of *C. tropicalis* almost 2.5 times more frequently than was the case in the control group. Interestingly, children in remission demonstrated the prevalence of *C. tropicalis* that was significantly decreased and comparable to the control group (Figure 2). The observed changes in the prevalence of *C. tropicalis* are similar to those in previously published reports. Hoarau et al. [18] also showed a significantly higher incidence of this yeast in the stool of patients with CD. Moreover, the researchers found a positive correlation between its presence and the concentration of ASCA antibodies. Apart from that, they established a link between the presence of *C. tropicalis* and increased colonization by the bacteria *E. coli* and *Serratia marcescens*. More extensive research into the matter revealed that these microorganisms form a thick layer of biofilm strongly adhering to the intestinal epithelium, which may promote an excessive immune response or cause intestinal mucosal barrier dysfunction, contributing to the formation of inflammatory lesions. Additionally, tests in animal models of IBD confirm the observations concerning the participation of *C. tropicalis* in the induction of the disease. CLEC7A knockout (resulting in a deficiency of the Dectin-1 receptor, which recognizes fungi) mice were characterized by an increased immune reaction in response to supplementation with *C. tropicalis* fungi. As a result, overproduction of pro-inflammatory cytokines, TNF-α, IFN-γ and IL-17, took place and intensification of inflammatory changes in the intestines followed [19]. Similar relationships were observed in the rat model of the disease. The rodents with colitis induced by the administration of trinitrobenzenesulfonic (TNBS) acid exhibited intensification of the inflammatory changes and increased synthesis of IL-1β and TNF-α, following having been infected with fungi of the genus *Candida* [20]. Moreover, our previous studies revealed that children with CD in the period of exacerbation of the disease exhibited significantly higher numbers of *Candida* spp. compared to the healthy control group. Interestingly, after the application of therapy and when the patients entered remission, the numbers of these yeasts decreased significantly [21].

We observed an increased prevalence of *C. neoformans* and *Malassezia* spp. in patients with both CD groups (Figure 2) compared to the control group; however, these changes were not statistically significant and need further study. There is little data on the participation of fungi of the genus *Malassezia* and *C. neoformans* in the course of IBD, but the reports published to date indicate an increased frequency of these species among patients, which is in line with our observations. Limon et al. [22] showed that the species *Malassezia restricta* was significantly more common in patients with CD than in healthy subjects, and its presence was associated with exacerbation of colitis in mice. While Liguori et al. [23] found that mucosal sections taken from the colon of people suffering from Crohn’s disease exhibited a more frequent occurrence of *Malassezia globosa* than the biopsies collected from healthy individuals. Furthermore, Li et al. [24] demonstrated differences between biopsies from the same patient, which were collected from inflamed sites vs. sites without evidence of inflammation; *C. neoformans* and *M. restricta* were overrepresented in the inflamed mucosa. In the aforementioned studies, only the presence of these species was analyzed, but no quantitative research was performed. In our study, quantitative evaluation showed that the number of fungi of the genus *Malassezia* was the lowest in the group of patients with newly diagnosed CD, being in the period of active disease. It is different, in patients in remission, who were diagnosed with CD several years prior to recruitment into the study, the number of these yeasts increased significantly (Figure 3). Such a trend, reversed in relation to the examination of prevalence, can be explained by the fact that the qualitative analysis did not include the samples that were negative in terms of the presence of the tested microorganisms, which could have influenced the results. On the other hand, this may suggest that quantitative changes within this genus are a secondary effect of the treatment that was administered, chronic inflammation, or the antibiotic therapy applied. As shown in several studies, the employed treatment and/or change in eating habits may significantly affect the changes in the gut mycobiome [21,25,26,27,28,29].

Data pointing to differences in the gut mycobiome between patients in the active phase of the disease vs. patients in remission (observed in this study as well) suggest that fungal dysbiosis may be involved in inflammation and disease induction, or is the result of chronic inflammation [21,24,25]. It seems interesting that the initial endeavors to support standard therapy with antifungal preparations have so far yielded promising results. Giving the mice an antifungal drug (fluconazole) resulted in alleviating the symptoms of the disease [19], while the application of antifungal drugs in a group of pediatric patients with UC contributed to a significant decrease in the UC activity rate, alleviation of clinical symptoms and improvement of the endoscopic and histological images [20].

Research limited to the participation of archaea in IBD has not yet provided definite results. In our study, we have not observed statistical differences in the predominance of archaea between groups. However, it is worth noting that *M. smithii* was the most common and most numerous in the group of children with active CD. In turn, in the group of patients in remission the amount of this methanogen DNA decreased (Figure 4). Results different from ours were obtained by Scanlan et al [30]. These researchers found that the presence of methanogens is less likely to be found in patients with IBD than in healthy people. Among patients with CD, the number of individuals positive for methanogens oscillated at around 30%, among patients with UC at 24%, while in the healthy control, the presence of methanogens was found in 50% of the examined individuals. Furthermore, Blais-Lecours et al. [31] detected that the species *M. smithii* was occurring with the same frequency in both the afflicted and healthy people, while the immunogenic species *M. stadtmanae* was three times more often found in people with IBD. Such varying results probably stem from different ages of the patients under examination. Our study covered children and adolescents up to 18 years of age, while the mean age of patients in the studies mentioned above was >40 years; what is more, they had been suffering from their ailments for many years. Already in the early 1980s, it was established that the production of methane escalates with age, which is later explained by the increase in the prevalence of methanogens in elderly people [32]. Perhaps age is the factor that determines the composition and number of archaea. Whereas the differences detected between patients with IBD and the control group later on result from the impact of the disease, long-term inflammatory process in the gastrointestinal tract, the therapy employed, changes in diet, or are a result of bacterial/fungal dysbiosis, which creates an optimal environment for the development of individual species of archaea. Results similar to ours were reported by Chechoud et al [33], who, similar to this study, took into consideration pediatric patients and did not observe significant differences between the presence of archaea in children with IBD compared to healthy children. Importantly, the investigators demonstrated that archaea were detected much less frequently in children than in adults [33]. These observations seem to confirm the assumptions that the composition of the “archaeobiome” is to a large extent determined by age, while the changes observed in adult patients with IBD are probably a secondary effect of the disease, but not the factor inducing the disease. Perhaps archaea contribute to the reappearance of exacerbations or support inflammation in adult patients; however, on the basis of such a small number of studies and non-optimized detection methods, it is not possible to clearly outline their role in the course of inflammatory bowel diseases. 

Although there is more and more evidence that autoimmune diseases are a consequence of a maladaptive immune system, mediated by the gut microbiota, the specific microbe responsible for the development of IBD has not been determined yet. Numerous studies expose changes concerning the composition of fungi and bacteria in patients with IBD; however, they do not give a clear answer as to whether the observed dysbiosis is a factor inducing the disease in genetically predisposed individuals or if it is only a secondary effect of the disease. The role of archaea is yet unknown, but the observed changes in their composition make one take a broader look at these microorganisms in order to determine their exact role in human health and disease.

In conclusion, the results presented above were obtained using the qPCR method, which allows for a precise quantification of selected species of microorganisms. The use of next-generation sequencing (NGS) would allow for a much more complete taxonomic picture of the mycobiome and archeobiome, but in this work we focused on the species most often described in the literature.

## 4. Materials and Methods

### 4.1. Patients

Pediatric patients aged 2 to 18 years with confirmed Crohn’s disease were recruited for the study at the University Children’s Hospital in Krakow. The diagnoses were made on the basis of the clinical picture, as well as endoscopic, histopathological and radiological examinations, according to the revised Porto criteria [34]. The study protocol was approved by the Jagiellonian University Bioethics Committee (No. 1072.6120.21.2020) on 23 January 2020. Patients were recruited into two research groups, which were compared to the control group. The first group consisted of children with newly diagnosed CD and disease activity assessed according to the PCDAI score >10 points. The second group included children with previously diagnosed CD who were in clinical remission at the moment of inclusion into the study (PCDAI ≤ 10 points). 

The inclusion criteria were as follows: age from 2 to 18 years; diagnosis of CD; informed consent to participate in the study signed by the patients’ parents or legal guardians or by the patients themselves if they were over 16 years of age. The exclusion criteria included: lack of an established diagnosis; antibiotic therapy up to 3 months prior to entering the study; the use of probiotic preparations up to 3 months before entering the study; confirmed lactose and gluten intolerance; irritable bowel syndrome; immunodeficiencies and neoplastic diseases. The control group included healthy children, not treated with antibiotics or supplemented with probiotics at least in the 3 months before the start of the study. Volunteers for this group were recruited in a non-hospital setting.

All patients with CD had their basic blood count parameters and biochemical examinations determined and, additionally, fecal samples were collected in order to evaluate selected groups of *Archaea* and yeast fungi. On the basis of the clinical picture and test results, PCDAI was assessed. All biochemical analyses were conducted at the University Children’s Hospital in Krakow. Stool samples were delivered under ultra-low freezing conditions (−80 °C) in sterile containers to the Department of Microbiology, Jagiellonian University Medical College, where DNA was isolated and further analyses were carried out.

### 4.2. DNA Extraction from the Stool Samples

The fecal samples were thawed and 150 mg of each material were precisely weighed (to determine the number of microbial cells in 1 g of stool at a subsequent stage). Isolation of fungal and archaeal DNA was carried out using an increased efficiency Genomic Mini AX Stool kit (A&A Biotechnology, Gdansk, Poland) along with our previously described modification [3,21,25] involving enzymatic lysis with lysozyme (Sigma, Saint Louis, MO, USA), mutanolysin, lyticase (A&A Biotechnology, Gdansk, Poland ) and buffer with β-mercaptoethanol (Sigma, Saint Louis, MO, USA), as well as mechanical lysis using the FastPrep homogenizer (MP Biomedicals, Irvine, CA, USA). 

### 4.3. Quantitative Real-Time PCR (qPCR):

**Figure 5:**
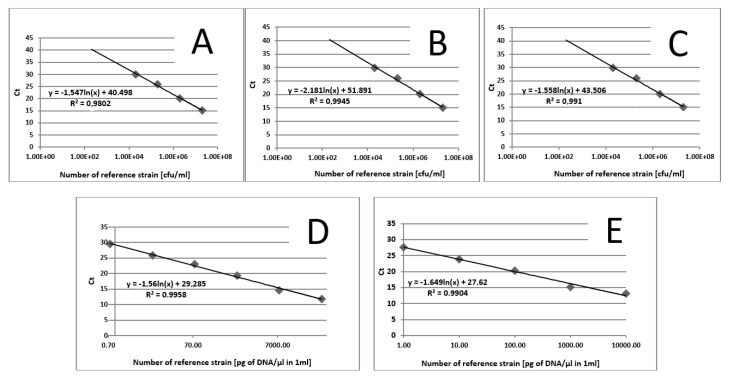
Standard curves generated from known numbers for respective microorganisms: (**A**)—*C. tropicalis*, (**B**)—*C. neoformans*, (**C**)—*Malassezia* spp., (**D**)—*M. smithii*, (**E**)—*M. stadtmanae*.

The samples obtained through the isolation process were tested for the presence of the following fungi: *Malassezia* spp., *C. tropicalis*, and *C. neoformans*, and two methanogenic species of archaea: *M. stadtmanae* and *M. smithii*. These particular species were selected as they were frequently found in fecal samples and due to their representativeness as well as the possibility to make references to the work of other researchers. Detection was conducted using *Taq*Man probes with the FAM reporter dye (Genomed, Warszawa, Poland). The composition of the reaction mixture was as follows: 2 μL DNA, 5 μL Real-Time PCR Mix Probe (A&A Biotechnology, Gdansk, Poland), 0.2 μL primers each (20 mM, Genomed, Warsaw, Poland), 0.6 μL *Taq*Man probe (10 mM, Genomed, Warsaw, Poland), 2 μL water (A&A Biotechnology, Gdansk, Poland). The probe and primer sequences used are summarized in Table 1. After preparing the reaction mixture, the samples were transferred into optical strips dedicated to CFX96 thermal cycler (BioRad, Hercules, CA, USA). All DNA samples were tested in duplicate. The reactions were supplemented with negative control containing water instead of DNA and positive control containing the DNA of the appropriate reference strain of the species studied (*M. furfur* ATCC 14521, *C. tropicalis* ATCC 13803, *C. neoformans* ATCC 204092, *M. smithii* DSM 861, *M. stadtmanae* DSM 3091). The reactions were conducted in accordance with the thermal programs set out in Table 1. The point at which the amplification signal was read was the reaction cycle in which the product growth reached the established cycle threshold (Ct) cutoff. Standard curves were generated from 10-fold serial dilutions (10^1^ to 10^7^ CFU/mL—Colony Forming Unit/mL) of every reference strain of fungi and from 10-fold serial dilutions of reference archaeal DNA. With the established Ct point on the standard curve, the numbers of particular microorganisms or their DNA concentrations were calculated in positive samples (Figure 5).

### 4.4. Statistical Analysis

Analyses of nominal variables were performed using the Chi square test. The Kruskal–Wallis test was applied to compare the numbers of fungal and archaeal cells in all groups under study. In order to determine the significance between particular groups, a post-hoc test of multiple comparisons of mean ranks was used for all groups. The result was deemed statistically significant when *p* < 0.05. Correlation between the variables was assessed using the Spearman’s rank correlation coefficient. All statistical analyses were carried out using the Statistica 13.3 software (StatSoft Inc., Tulsa, OK, USA).

## Figures and Tables

**Table 1:** Real-time PCR primer/probe sequences and PCR parameters for detecting respective microorganisms.

*Primer and Probe*	Sequence (5′ to 3′)	Thermal Cycling Programme	References
***C. tropicalis*** Forward primer Reverse primerProbe	GCGGTAGGAGAATTGCGTT TCATTATGCCAACATCCTAGGTTTA 6-FAM-CGCAGTCCTCAGTCTAGGCTGGCAG-BHQ-1	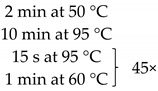	[35]
***C. neoformans*** Forward primer Reverse primerProbe	GCCGCGACCTGCAAAG GGTAATCACCTTCCCACTAACACAT 6-FAM-ACGTCGGCTCGCC-BHQ-1	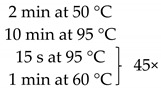	[36]
***Malassezia spp.*** Forward primer Reverse primerProbe	GTAGACTCCATCTAAAGCTAAAT CTTTTAACTCTCTTTCCAAAGT 6-FAM-CCCTCACGGTACTTGTTCGCT-BHQ-1	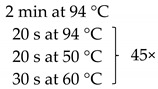	[37]
***M. smithii*** Forward primer Reverse primerProbe	CCGGGTATCTAATCCGGTTC CTCCCAGGGTAGAGGTGAAA 6-FAM-CCGTCAGAATCGTTCCAGTCAG-BHQ-1	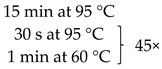	[38]
***M. stadtmanae*** Forward primer Reverse primerProbe	AGGAGCGACAGCAGAATGAT CAGGACGCTTCACAGTACGA 6-FAM-TGAGAGGAGGTGCATGGCCG-BHQ-1	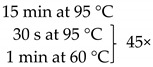	[38]

## Data Availability

The data presented in this study are not publicly available as a matter of confidentiality. However, these data are available upon request from the corresponding author.
